# MLX plays a key role in lipid and glucose metabolism in humans: Evidence from in vitro and in vivo studies

**DOI:** 10.1016/j.metabol.2023.155563

**Published:** 2023-07

**Authors:** Shilpa R. Nagarajan, Eilidh J. Livingstone, Thomas Monfeuga, Lara C. Lewis, Shahul Hameed Liyakath Ali, Anandhakumar Chandran, David J. Dearlove, Matt J. Neville, Lingyan Chen, Cyrielle Maroteau, Maxwell A. Ruby, Leanne Hodson

**Affiliations:** a Oxford Centre for Diabetes, Endocrinology and Metabolism, Radcliffe Department of Medicine, University of Oxford, Churchill Hospital, Oxford, UK; b Novo Nordisk Research Centre Oxford, Innovation Building, Oxford, UK; c National Institute for Health Research Oxford Biomedical Research Centre, Oxford University Hospital Trusts, UK

**Keywords:** MLX, Hepatocytes, DNL, Human, Glucose, Insulin

## Abstract

**Background and aim:**

Enhanced hepatic de novo lipogenesis (DNL) has been proposed as an underlying mechanism for the development of NAFLD and insulin resistance. Max-like protein factor X (MLX) acts as a heterodimer binding partner for glucose sensing transcription factors and inhibition of MLX or downstream targets has been shown to alleviate intrahepatic triglyceride (IHTG) accumulation in mice. However, its effect on insulin sensitivity remains unclear. As human data is lacking, the aim of the present work was to investigate the role of MLX in regulating lipid and glucose metabolism in primary human hepatocytes (PHH) and in healthy participants with and without *MLX* polymorphisms.

**Methods:**

PHH were transfected with non-targeting or *MLX* siRNA to assess the effect of *MLX* knockdown on lipid and glucose metabolism, insulin signalling and the hepatocellular transcriptome. A targeted association analysis on imputed genotype data for *MLX* on healthy individuals was undertaken to assess associations between specific *MLX* SNPs (rs665268, rs632758 and rs1474040), plasma biochemistry, IHTG content, DNL and gluconeogenesis.

**Results:**

*MLX* knockdown in PHH altered lipid metabolism (decreased DNL (*p* < 0.05), increased fatty acid oxidation and ketogenesis (*p* < 0.05), and reduced lipid accumulation (*p* < 0.001)). Additionally, *MLX* knockdown increased glycolysis, lactate secretion and glucose production (*p* < 0.001) and insulin-stimulated pAKT levels (*p* < 0.01) as assessed by transcriptomic, steady-state and dynamic measurements. Consistent with the in vitro data, individuals with the rs1474040-A and rs632758-C variants had lower fasting plasma insulin (*p* < 0.05 and *p* < 0.01, respectively) and TG (*p* < 0.05 and *p* < 0.01, respectively). Although there was no difference in IHTG or gluconeogenesis, individuals with rs632758 SNP had notably lower hepatic DNL (*p* < 0.01).

**Conclusion:**

We have demonstrated using human in vitro and in vivo models that MLX inhibition favored lipid catabolism over anabolism and increased glucose production, despite increased glycolysis and phosphorylation of Akt, suggesting a metabolic mechanism that involves futile cycling.

## Highlights

• We investigated the role of MLX in human liver metabolism in vivo and in vitro.
• MLX knockdown reduced lipid content by stimulating fatty acid oxidation in vitro.
• MLX knockdown increased glucose production, despite higher pAKT levels.
• Humans with MLX mutations have lower fasting plasma insulin, triglycerides and DNL.
• MLX modulation shifts cellular metabolism from anabolic to catabolic processes.

## Graphical abstract

**Figure:**
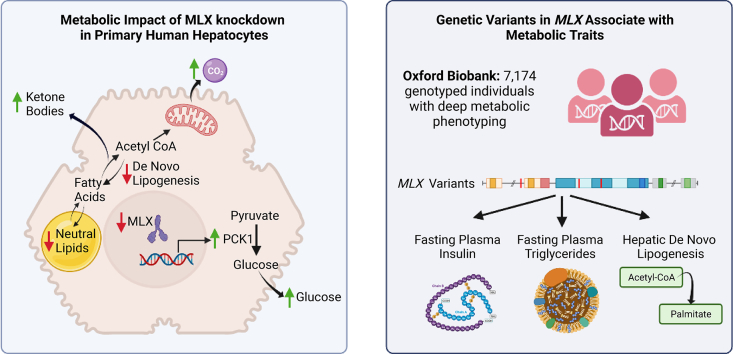

## 1 Introduction

Non-alcoholic fatty liver disease (NAFLD) is defined by the pathological accumulation of intrahepatocellular triglyceride (IHTG) (>5 % of liver weight) and is often associated with obesity and type 2 diabetes (T2D) [1]. The prevalence of T2D in patients with NAFLD is two-fold greater than in the general population and is implicated in accelerating NAFLD progression [2]. Despite the few antidiabetic drugs in phase 4 clinical trials proposed to reduce both hyperglycaemia and hepatic steatosis, there is currently no pharmacotherapy approved for NAFLD and limited effective pharmacotherapies that are suitable for patients diagnosed with both NAFLD and T2D [3].

Studies have suggested that the underlying mechanism for the pathogenesis of NAFLD and insulin resistance is the shift in cellular metabolism away from β-oxidation towards fatty acid esterification, which is primarily driven by increased de novo lipogenesis (DNL) [4]. Thus, DNL has often been suggested as a key pathway to target for drug development to treat NAFLD. The two main transcription factors that regulate DNL are sterol-regulatory element-binding protein 1c (SREBP-1c) and carbohydrate regulatory element-binding protein (ChREBP); both activating key enzymes including ACC, FAS and SCD1 [4]. While SREBP-1c and ChREBP are directly upregulated by insulin and glucose, respectively. ChREBP is also indirectly regulated by insulin (via LXRs) and stimulates liver pyruvate kinase, a regulatory enzyme for glycolysis and gluconeogenesis, making ChREBP an attractive drug candidate [5].

Studies investigating the effects of ChREBP modulation have mainly been performed in mice or mouse hepatocytes and present with mixed results [6], [7], [8], [9], [10]. While some studies have shown beneficial effects of liver-specific inhibition of ChREBP for both IHTG content and whole-body insulin sensitivity in ob/ob mice [7], the majority of studies suggest improved IHTG content in hepatic steatosis only. For example, ChREBP-deficient mice fed a high starch diet had a 65 % reduction in DNL (measured via incorporation of ^2^H_2_O into plasma palmitate) and lower IHTG content despite becoming hyperinsulinaemic and mildly hyperglycaemic [8], [10], [11], while overexpression of ChREBP led to 3-fold greater IHTG content but improved glucose tolerance [6], [11]. This may be due to functional differences in ChREBP between isoforms (ChREBPα (canonical) vs. ChREBPβ) and tissues (e.g. adipose vs. liver) [12]. Therefore, modulating the expression of a compulsory binding partner of ChREBP in hepatocytes, such as the coactivator Max-like protein factor X (*MLX*), may help resolve its potential for pharmacological modulation.

Few studies thus far have explored the role of MLX in regulating hepatic metabolism. Adenoviral delivery of a dominant negative form of *MLX* (i.e. inhibitory) in the liver of C57BL/6 J mice reduced IHTG content and improved glucose intolerance [9]. In humans, a meta-analysis including 81,412 individuals with T2D of diverse ancestry identified *MLX* (p.Gln139Arg rs665268) as one of five T2D association signals [13]. However, the role of human MLX in modulating hepatic lipid and glucose metabolism has not previously been investigated. Therefore, the aim of the present study was to investigate the metabolic function of MLX in vitro (utilising primary human hepatocytes (PHH)) and in vivo in healthy human participants.

## 2 Methods

### 2.1 Oxford Biobank

Oxford Biobank (OBB) collected data on ∼8000 healthy participants living in Oxfordshire, of ages 30–50 on enrolment [14]. Past medical history, anthropometric measurements, body composition assessed using dual energy X-ray absorptiometry and biological samples for DNA and cardiovascular and obesity related biochemical measures, were obtained at screening. OBB was granted ethical approval by the Oxfordshire Clinical Research Ethics Committee (18/SC/0588).

### 2.2 Genotyping

Genotyping in the OBB was performed using the UK Biobank Axiom array (847,441 genome-wide genotyped markers) for 7174 individuals, and from this 72,355,667 variants imputed against UK10k haplotype reference panel, merged with the 1000 Genomes Phase 3 reference panel, using the IMPUTE v2 software (https://mathgen.stats.ox.ac.uk/impute/impute_v2.html). We used VCFtools v 0.1.13 to extract genotypes of interest then converted to PLINK (v1.90b3e 64-bit) format using a linear regression sex adjusted additive model. A 7 kb genomic region of chromosome 17 encompassing the *MLX* gene (40,718,300 bp–40,725,300 bp, GRCH37) containing 21 single nucleotide polymorphisms (SNPs) from the OBB imputation dataset was interrogated against log transformed BMI, waist, hip, DEXA fat mass, glucose, insulin, HOMA-IR and lipid phenotypes. The study focused on 3 SNPs in MLX: rs665268, rs1474040, and rs632758. The SNPs rs665268 and rs1474040 were available as chip genotypes data on the Affimetrix Uk Biobank 850 k Chip, whereas rs632758 was imputed from that chip data. The INFO data from the rs632758 imputation gave an R2 = 0.9995 and the “best guess genotype” was used with an additional filter for the estimated posterior probabilities for individual genotypes for values falling below 0.8 to be counted as missing data. All analyses were adjusted for age and first 4 principal components. Glucose, insulin, HOMA-IR and lipid phenotypes were also adjusted for BMI, waist and hip with or without BMI, android and gynoid DEXA fat mass with or without percentage fat mass. The genotype associations are presented as geometric mean ± 95 % confidence intervals.

### 2.3 Measurement of DNL, gluconeogenesis and IHTG content: an exploratory study

Retrospective data from a selected number of participants previously recruited for other studies utilising stable isotope methodology (published [15], [16], [17], [18], [19], [20] and unpublished) provided the opportunity to assess the effect of *MLX* SNPs on hepatic DNL, gluconeogenesis and IHTG content in humans. Healthy participants (i.e. free from any known disease, medications known to affect glucose or lipid metabolism, and excessive alcohol consumption) were recruited from the OBB [14]. All participants had consumed deuterated water the evening prior to the study day (^2^H_2_O; 3 g/kg body water), followed by overnight fasting and collection of fasting blood samples the next morning. Percentage of hepatic DNL was assessed based on the incorporation of deuterium from ^2^H_2_O into VLDL-TG palmitate using GC–MS; monitoring ions with *m*/*z* 270 (M + 0) and 271 (M + 1) [21]. Similarly, percentage of gluconeogenesis was determined based on the incorporation of deuterium from ^2^H_2_O into glucose extracted from plasma using GC–MS [22]; monitoring ions with *m*/*z* 319 (M + 0) and 320 (M + 1). IHTG percentage was measured using magnetic resonance spectroscopy (^1^H-MRS) as previously described [23].

### 2.4 Primary human hepatocyte cell culture

Cryoplatable PHH (BioreclamationIVT, M00995-P and F00995-P; or Lonza, HUCPI) were seeded into collagen-coated 96-well plates (Perkin Elmer collagen-coated CellCarrier-96 Ultra, 6055708). 24 h later, PHH were washed once with DPBS (ThermoFisher, 14190250) and maintained in modified 5C PHH maintenance medium (5C media) [24], this was refreshed every 2–3 days.

### 2.5 siRNA transfection in 96-well and 12-well format

PHH were transfected 7 days post seeding with a final concentration of 30 nM siRNA, and 0.3 μl RNAiMAX (Invitrogen, 13778-150) in OptiMEM (Gibco, 31985062), then maintained in 5C media. siRNAs used were purchased from Dharmacon: *MLX* (L-009724-00-0005), *DGAT2* (L-009333-00-0005) and Non-targeting (NT) (D-001810-10-20).

### 2.6 Free fatty acid (FFA) mixture

One-week post transfection, PHH were treated with 5C media containing either 0 μM or 800 μM FFA mix diluted in 5C media for 72 h. FFA mix was produced as described previously [25], with a modified FFA ratio for oleic acid (Sigma, O1008), palmitic acid (Sigma, P0500), linoleic acid (Sigma, L1376), and α-linolenic acid (Sigma, L039) of 45:30:24:0.01.

### 2.7 Starvation and glucose production assay

To measure PHH glucose production, PHH were transfected with siRNA for nine days then starved in low glucose DMEM media (Gibco, 110540-20), supplemented with 1× Glutamax (Gibco, 35050-038) and 0 μM or 800 μM FFA mix for 19 h. After washing trice in PBS, PHH were fed gluconeogenesis media, final concentrations are shown: glucose free medium (Thermofisher, A1443001), lysine (2 mM, L9037, Sigma Aldrich), lactate (2 mM, L7022-50G, Sigma-Aldrich), sodium pyruvate (2 mM, gibco, 11360-039), glutamine (2 mM, gibco, 25030-024), HEPES (15 mM, gibco, 15630-080) and forskolin (0.015 mM, Sigma, F3917-25MG). To measure the contribution of gluconeogenesis and glycogenolysis to total glucose production, powdered glucose-free DMEM (D5030-10X1L, Merck) reconstituted in ^2^H_2_O was used instead of the standard premade glucose-free media.

After the 5-hour incubation in gluconeogenesis media, PHH were fixed in 4 % FA (Sigma, 47,608) for 10 min, or lysed for subsequent assays, as required. Supernatant was collected for the glucose production or albumin secretion assay. Secreted albumin was measured using a human serum albumin HTRF kit (Cisbio, 6FHSAPEG), following manufacturer's protocol. Glucose was measured using the Glucose-Glo detection assay (Promega, J6022), following manufacturer's instructions. Luminescence was measured using a BMG CLARIOstar Plus.

### 2.8 Measurement of glucose produced from gluconeogenesis or glycogenolysis

Cellular gluconeogenesis in PHH was assessed by the appearance of labelled glucose (from ^2^H_2_O) in the gluconeogenesis media. Briefly, media was deproteinised with ethanol and centrifuged with the supernatant being collected and dried prior to methylhydroxylamine hydrochloride in pyridine (2%w/v) being added. Samples were heated and once cooled, BSTFA + 1 % TCMS was added and samples were heated again, before being cooled, dried, and then reconstituted in decane. Tracer enrichment in media glucose was analysed by GC-mass spectrometry with selected ion monitoring [26] and data were analysed using quantitative mass spectral analysis (QMSA) based on the method described by Tayek and Katz [27]. Fractional gluconeogenesis was calculated using the “average” method described by Chacko et al. [22] and data was normalised to cell protein content.

### 2.9 Nile red lipid accumulation assay

Fixed PHH were stained with Nile Red (1 μM, Sigma, N1142) for 1 h at room temperature (RT) then washed with DPBS (ThermoFisher, 14,190,250). Fluorescence was measured on a BMG ClARIOstar Plus. The Nile Red ratio was calculated by dividing the neutral lipid fluorescence (540–15 nm/600–20 nm) by the phospholipid fluorescence (540–15 nm/640–20 nm) [28].

### 2.10 Western blot

PHH were washed with cold DPBS (ThermoFisher, 14190250) prior to harvesting in RIPA buffer (ThermoFisher, Horsham, UK, 89901) supplemented with 1× protease inhibitor cocktail (ThermoFisher, 78,446). Protein concentration of cell lysates was determined using a BCA Protein Assay Kit (ThermoFisher, 23225). 20 μg protein was separated by SDS-PAGE gels (Bio-Rad, Horsham, UK, 4561094) and semi-dry transferred using iBlot 2 Dry blotting system (Invitrogen, IB21001). Blots were probed with anti-MLX (1:1000; CST D8G6W), rhodamine anti-tubulin (1:1000; Bio-rad 12004166) and goat anti-rabbit (1:10000; Invitrogen 65-6120) antibodies and then imaged using Chemi-doc (Bio-Rad).

### 2.11 Measurement of DNL and fatty oxidation

Total lipids were extracted from cell lysates according to the Folch method [29] and the micromolar quantities of each fatty acid were quantified using a 6890 Network Gas Chromatography (GC) System (Agilent Technologies; CA, USA) as previously described [30]. Intracellular DNL was based on the incorporation of ^13^C-labelled glucose and fructose in the media into fatty acids in intracellular TG. Ions with mass-to-charge ratios (*m*/*z*) of M + 0, M + 1, M + 2, M + 3, M + 4 and M + 5 were determined by GC-mass spectrometry (GC–MS) using a 5890 GC coupled to a 5973 N MSD (Agilent Technologies; CA, USA) and selected ion monitoring [21], [31]. To measure fatty acid oxidation in PHH, we measured the appearance of ^2^H_2_O using a Finnigan GasBench-II (ThermoFisher Scientific, UK) in cell media derived from the ^2^H-labelled palmitate [D_31_] and oleate [D_33_] in the media [32].

### 2.12 Biochemical analysis

For biochemical analysis of extracellular metabolites, media concentrations of TG, 3-hydroxybutyrate (3-OHB), glucose and lactate were measured on the AU480 Chemistry Analyzer (Beckman Coulter; High Wycombe, UK), which had previously been optimised for low concentrations found in vitro [30], and results were normalised to intracellular protein concentration. Intracellular levels of phosphorylated AKT were measured via homogenous time resolved fluorescence (hTRF) using a Phospho-AKT (Ser473) kit (Cisbio, #64AKSPET), according to manufacturer's instructions.

### 2.13 Seahorse assays

PHH were seeded into collagen coated XF96 cell culture plates (Agilent Technologies, 101085-004), maintained in 5C media and transfected with siRNA as before. Eight days post-transfection, either the Agilent Seahorse XF Glycolysis Stress Test Standard Assay (Agilent Technologies, 103020-100), or the Agilent Seahorse XF MitoStress Test (Agilent Technologies, 103015-100), were performed following the manufacturer's instructions, using the ‘constant concentration’ volumes for loading. Plates were analysed in a Seahorse XFe 96 Analyzer (Agilent Technologies). Afterwards, Hoechst 33342 (62249) fluorescence intensity measured in a BMG CLARIOstar Plus was used for normalisation. Data was analysed using the Wave software (version 2.6.3.5, Agilent Technologies).

### 2.14 RNA extraction and qPCR

RNA extraction was performed with Dynabeads mRNA DIRECT Purification Kit (Invitrogen, 61012). DNase treatment and reverse transcription was performed using SuperScript IV VILO Master Mix with ezDNase Enzyme (Invitrogen, 11766500). All procedures followed the respective manufacturer's protocols. qPCR assays were run in a 384-format on the Biorad CFX384, using TaqMan Fast Advanced Master Mix (Invitrogen, 4444557) and TaqMan probe (*MLX* (Hs00538258_M1), TBP (Hs00427620_M1)). The ΔΔC(T) method was used to calculate the relative abundance of transcripts.

### 2.15 RNA-sequencing data generation

mRNA was extracted from the PHH 10 days post-transfection (3 independent experiments with cells from a different donor in each) and used to prepare sequencing libraries with a QuantSeq 3′mRNA-Seq Library Prep Kit-FWD (Cat.no. 15, Lexogen). Final libraries were pooled and run on an Illumina NextSeq 500 system after assessing their quality using an Agilent 4200 Tapestation system. Sequenced reads were processed with Trim Galore! (v. 0.6.4_dev) and then aligned to a decoy-aware index (reference: Gencode GRCh38, release 38) with Salmon (v.1.9.0; arguments used: –noLengthCorrection and –numBootstraps 100) [33]. Quality control and alignment metrics were obtained using FastQC (v.0.11.9) and MultiQC (v. 1.11) [34]. After excluding one sample (si*MLX* group) due to insufficient sequencing depth, the average mapping rate and number of reads were 77.7 % (s.d. 4.7 %) and 2.9 M (s.d. 0.4), respectively. The final sample size included in analyses were n = 14 si*MLX* and n = 15 siNT.

### 2.16 RNA-sequencing analyses

All data processing and analyses were performed using R (v. 4.2.1). Details about packages versions and function parameters are available in the github repository (cf. Data availability section). RNA-seq data was prepared and analysed using Wasabi and Sleuth [35], and summarized at the gene-level using annotations from the annotable package. Sleuth's Wald test was used to perform differential expression analyses, correcting for PHH donor-specific effects. Enriched KEGG pathways were obtained with the Clusterprofiler package [36]. The Benjamini-Hochberg procedure was used for adjusting *p*-values for false discovery rate in all sequencing data-based analyses.

Genes potentially regulated by MLX binding were determined using CHIP-seq data targeting *MLX* in HepG2 cells from the ENCODE portal [37], the ChIPpeakAnno package [38] and annotations from EnsDb.Hsapiens.v86. Peaks were mapped to genes whose transcription start site (TSS) was located within 1500 bp either direction, restricting to genes also included in the differential expression analysis. Potential MLX targets were then ranked by signal value, which corresponds to the statistical significance of the peak call and reflects the likeliness of protein-DNA binding events. After selecting a set of genes made of top 50 *MLX* targets, a gene-set enrichment analysis was carried out using the fgsea package [39] alongside other sets of predicted transcription factor targets (“tft.gtrd” MSigDB geneset from the package msigdf) [40]. To generate a heatmap, the count data was first batch-corrected to account for the donor effects using Combat_Seq from the sva package [41], followed by a variance-stabilizing transformation (VST) with DESeq2 [42] and scaled per gene. Finally, the ComplexHeatmap's package was used for plotting and clustering [42].

### 2.17 Data availability

The CHIP-seq data analysed is publicly available on the ENCODE portal (encodeproject.org; experiment ENCSR125DAD, file ENCFF132AJP). The sequencing data generated has been deposited in the Gene Expression Omnibus (GEO) under the accession GSE219162. The R code used to process this data and generate the related figures is available on the github repository: novonordisk-research/MLX_siRNA_RNAseq.

### 2.18 Statistical analysis

Data are presented as the mean ± standard error (SEM) for all except the genotype data in Table 1 and Fig. 4. GraphPad Prism 9.0.1 was used for statistical analysis. To control for donor and batch variability of PHH, all data was normalised to siNT 0 μM FFA. A *t*-test was used for the analysis of two variables and two-way ANOVA with the Geisser-Greenhouse correction and Dunnett's multiple comparisons test was performed for comparison of multiple groups. Statistical significance was set at *p ≤ 0.05, **p ≤ 0.01, ***p ≤ 0.001.

**Table 1:** Participant characteristics for genotype subgroups in MLX SNPs rs1474040, rs632758 and rs665268. Age, BMI, HOMA-IR and DEXA data are represented as geometric means ± 95 % confidence interval. *p* values are calculated based on a sex combined additive linear regression model adjusted for sex, calculated in the PLINK software. DEXA analyses are adjusted for age and the first 4 principal components. HOMA-IR is adjusted for age, first 4 principal components and BMI. Statistical significance was set at *p* < 0.05.

	rs632758				rs665268				rs1474040			
	A/A	C/A	C/C	*p*	A/A	G/A	GG	*p*	G/G	A/G	A/A	*p*
*n* =	2247	3422	1375	–	3698	2787	561	–	3955	2622	470	–
M/F	990/1257	1493/1929	584/791	ns	1603/2095	1212/1575	252/309	ns	1753/2202	1107/1515	208/262	ns
Age	41.6 ± 0.1	41.6 ± 0.1	41.6 ± 0.2	ns	41.6 ± 0.1	41.7 ± 0.1	41.5 ± 0.3	ns	41.5 ± 0.1	41.6 ± 0.1	41.7 ± 0.3	ns
BMI (kg/m^2^)	25.59 ± 0.2	25.38 ± 0.1	25.46 ± 0.2	ns	25.69 ± 0.4	25.41 ± 0.2	25.43 ± 0.1	ns	25.41 ± 0.4	25.43 ± 0.1	25.46 ± 0.1	ns
Waist cm	85.88 ± 0.5	85.80 ± 0.4	85.80 ± 0.7	ns	85.71 ± 0.43	85.80 ± 0.43	86.75 ± 1.03	ns	86.06 ± 0.43	85.54 ± 0.5	85.54 ± 1.19	ns
Hip cm	100.89 ± 0.4	100.89 ± 0.3	101.19 ± 0.4	ns	100.99 ± 0.3	100.79 ± 0.3	101.09 ± 0.8	ns	100.99 ± 0.3	100.89 ± 0.3	100.79 ± 0.7	ns
HOMA-IR	**2.7** **±** **0.1**	**2.6** **±** **0.04**	**2.6** **±** **0.1**	**0.005**	2.6 ± 0.04	2.7 ± 0.05	2.8 ± 0.1	0.05	2.7 ± 0.04	2.6 ± 0.05	2.6 ± 0.1	0.1

DEXA												
*n* =	1425	2219	926		2424	2789	361		2554	1707	310	
M/F	612/813	971/1248	382/544		1037/1383	759/1030	169/192		1115/1439	720/978	131/179	
Fat mass kg	22.47 ± 0.45	22.25 ± 0.35	22.47 ± 0.55	ns	**22.03** **±** **0.34**	**22.47** **±** **0.40**	**23.38** **±** **0.98**	**0.004**	22.47 ± 0.33	22.24 ± 0.41	22.47 ± 0.92	ns
Android fat kg	1.728 ± 0.54	1.695 ± 0.43	1.709 ± 0.68	ns	**1.670** **±** **0.41**	**1.731** **±** **0.49**	**1.876** **±** **0.12**	**0.001**	1.723 ± 0.40	1.687 ± 0.49	1.718 ± 0.11	ns
Gynoid fat kg	3.839 ± 0.07	3.793 ± 0.06	3.866 ± 0.09	ns	3.797 ± 0.06	3.839 ± 0.06	3.905 ± 0.15	0.06	3.82 ± 0.05	3.816 ± 0.07	3.843 ± 0.15	ns
Android gynoid ratio	0.45 ± 0.01	0.447 ± 0.01	0.442 ± 0.01	ns	**0.44** **±** **0.01**	**0.451** **±** **0.01**	**0.481** **±** **0.02**	**0.0006**	0.451 ± 0.01	0.442 ± 0.01	0.447 ± 0.02	ns

## 3 Results

### 3.1 Hepatocyte-specific *MLX* knockdown does not affect PHH function

**Fig. 1:**
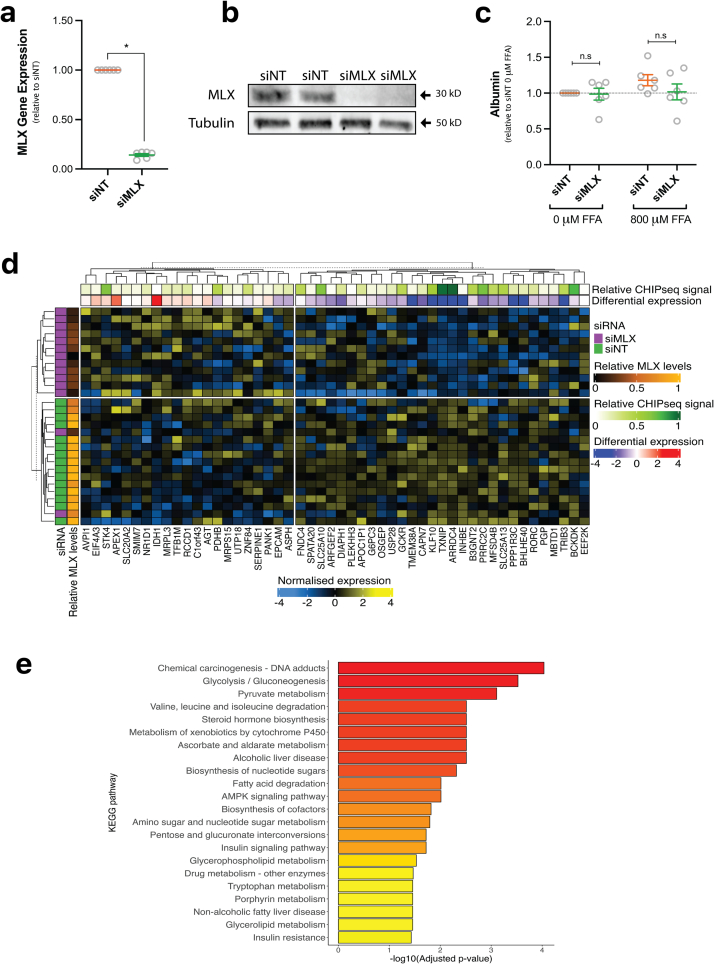
Changes in transcriptome but not hepatocyte function following *MLX* knockdown in PHH. Ten days following transfection with siNT or si*MLX* cells were lysed for quantification of **(a)** MLX gene and **(b)** protein expression. **(c)** Following transfection, 0 or 800 μM of FFAs were added to the media 24 h before media collection and albumin secretion was measured as a marker of hepatic function. Results are expressed as fold change over siNT or siNT 0 μM FFA and mean + SEM (n = 6; each dot represents a donor or independent experiment). *p ≤ 0.05, ** *p* ≤ 0.01, ***p ≤ 0.001 **(d–e)** Total RNA was extracted and sequenced to reveal transcriptional changes following knockdown, including (**d)** heatmap and clustering analysis based on the expression levels of the top 50 *MLX* target genes and **(e)** KEGG enrichment analysis for significant pathways. Heatmap annotation: the *MLX* normalised expression and CHIP-seq signal value (log transformed) were rescaled between 0 and 1; differential expression values are −log10(*p*-values) ∗ sign (log-fold changes).

To determine the impact of hepatocyte-specific *MLX* knockdown on lipid and glucose metabolism, PHH were treated with si*MLX* or a non-targeting control (siNT) for 10 days prior to functional measurements. There was a ~85 % reduction in *MLX* mRNA expression (*p* = 0.002) (Fig. 1a) and a ~81 % reduction in protein levels with si*MLX*-treatment compared to siNT (Fig. 1b). *MLX* knockdown had no effect on albumin secretion, suggesting hepatocyte function was unaffected (Fig. 1c). MLX is a compulsory binding partner for the transcription factors *MLXIPL* (ChREBP) and *MLXIP* (MondoA) and, upon heterodimer formation, it stimulates the transcription of downstream targets such as *TXNIP*. As expected, decreased *MLX* mRNA did not influence the expression of its primary binding targets, *MLXIPL* and *MLXIP* (data not shown), however it did decrease the expression of *TXNIP*, a canonical ChREBP target gene (Fig. 1d).

To determine the effect of knocking down *MLX* on the transcriptome, RNA-sequencing was performed on PHH after siRNA transfection. First, to verify that *MLX* knockdown affects expression of downstream targets of *MLX*, publicly available CHIP-seq data targeting *MLX* in HepG2 cells was used to determine the 50 genes with the most statistically significant binding of MLX within 1500 base pairs of their transcription start sites. A gene-set enrichment analysis was then performed and revealed that these 50 genes are the most significantly downregulated gene set (normalised enrichment score NES = −1.92, *p* = 2 × 10^−4^, adjusted *p* = 0.03) among MSigDB transcription factor gene sets (S1 Table 1). Clustering PHH samples based on the expression levels of these 50 targets largely segmented the treatment conditions (si*MLX* vs siNT) and their respective *MLX* levels (Fig. 1d). At the transcriptome-wide level, after filtering out low detected genes, a total of 200 genes were downregulated, and 110 genes upregulated (adjusted *p* ≤ 0.05; S1 Table 2). An overrepresentation enrichment analysis using KEGG pathway gene sets revealed that differentially expressed genes are mainly involved in metabolic-related pathways, such as glycolysis/gluconeogenesis, amino-acid metabolism and fatty acid degradation (Fig. 1e and S1 Table 3).

### 3.2 *MLX* knockdown decreased neutral lipid content and increased fatty acid oxidation in PHH

**Fig. 2:**
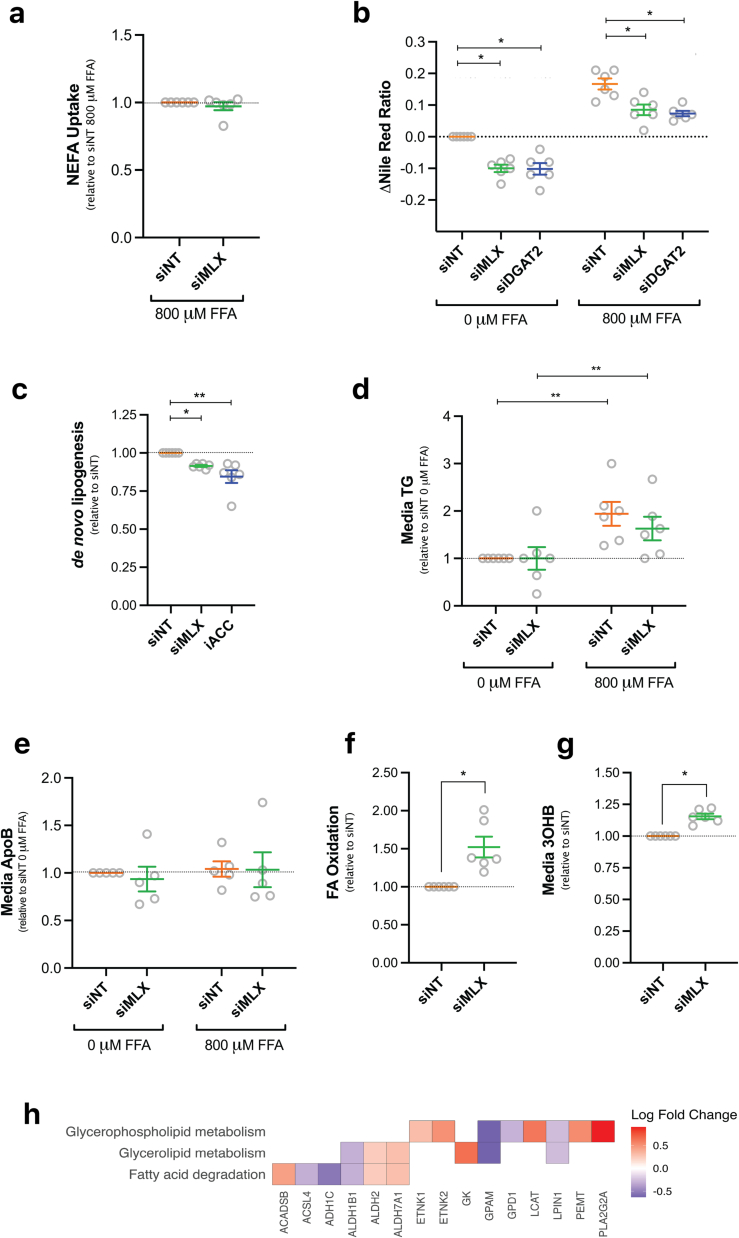
Neutral lipid reduction by stimulating FA oxidation and inhibiting DNL following *MLX* knockdown in PHH. Seven days following transfection with siNT or si*MLX* cell were treated for 72 h with 0 or 800 μM of FFAs before the measurement of **(a)** NEFA uptake **(d)** TG **(e)** ApoB and **(g)** 3OHB from media and **(b)** neutral lipid content (measured via Nile Red) from cell lysates. For the measurement of DNL, **(c)**^13^C labelled glucose and fructose were added at the time of FFA treatment and ^13^C enrichment into palmitate was quantified following lipid extraction. **(b–c)** Treatment with DGAT2 (using siRNA) and ACC (using CP-640186) inhibitors occurred alongside siNT and si*MLX* transfection. For the measurement of fatty acid (FA) oxidation, **(f)** deuterated FFAs were added for 72 h prior to media collection and enrichment into water was measured. **(h)** The RNA-sequencing data was used to highlight the significantly differentially expressed genes (adjusted *p*-value ≤ 0.05) in relevant affected KEGG pathways. Results are expressed as fold change over siNT or siNT 0 μM FFA and mean + SEM (n = 5–6; each dot represents a donor or independent experiment), *p ≤ 0.05, ** *p* ≤ 0.01, ***p ≤ 0.001. (For interpretation of the references to colour in this figure legend, the reader is referred to the web version of this article.)

To assess the role of *MLX* on intrahepatocellular lipid metabolism, siRNA-treated PHH were exposed to either 0 or 800 μM of a physiological mix of FFAs for 3 days prior to harvest. PHH took up ~70 % of FFAs in the media and this did not differ between siNT and si*MLX*-treated cells (Fig. 2a). In accordance with previously published animal data [9], *MLX* knockdown significantly lowered neutral lipid accumulation in the absence and presence of 800 μM FFA to a similar extent as knockdown of *DGAT2* (Fig. 2b). Reduced neutral lipid content in hepatocytes can be attributed to three main pathways: 1) reduced DNL 2) increased secretion of TG-enriched very-low density lipoprotein particles, and/or 3) increased fatty acid oxidation. Given the role of MLX in regulating downstream fatty acid synthesis, *MLX* knockdown was associated with reduced DNL, as expected (8 % reduction relative to siNT, *p* = 0.042) (Fig. 2c). In comparison, treatment with an inhibitor of ACC (rate-limiting enzyme in the DNL) resulted in a 15 % reduction (*p* = 0.001) in DNL (Fig. 2c). Media TG and apoB content were not significantly different between siNT and si*MLX*-treated cells (Fig. 2d–e), suggesting no difference in secretion. To measure the effect of *MLX* knockdown on fatty acid oxidation, siRNA-treated PHH were incubated with deuterated FFAs for 3 days and the appearance of ^2^H in media H_2_O was measured. *MLX* knockdown was associated with a 50 % increase in fatty acid oxidation compared to siNT (*p* = 0.031) (Fig. 2f). This was supported by a 16 % increase (*p* = 0.031) (Fig. 2g) in media 3OHB, suggesting an upregulation in ketogenesis or ‘incomplete’ oxidation in these cells. These results are consistent with the significant dysregulation of genes such as *GPAM* involved in glycerolipid metabolism (adjusted *p* = 0.04), glycerophospholipid metabolism (adjusted *p* = 0.03) and fatty acid degradation (adjusted *p* = 9.7 × 10^−3^) (Fig. 2h and S1 Table 3).

### 3.3 *MLX* knockdown results in greater glucose production, despite increased pAKT

**Fig. 3:**
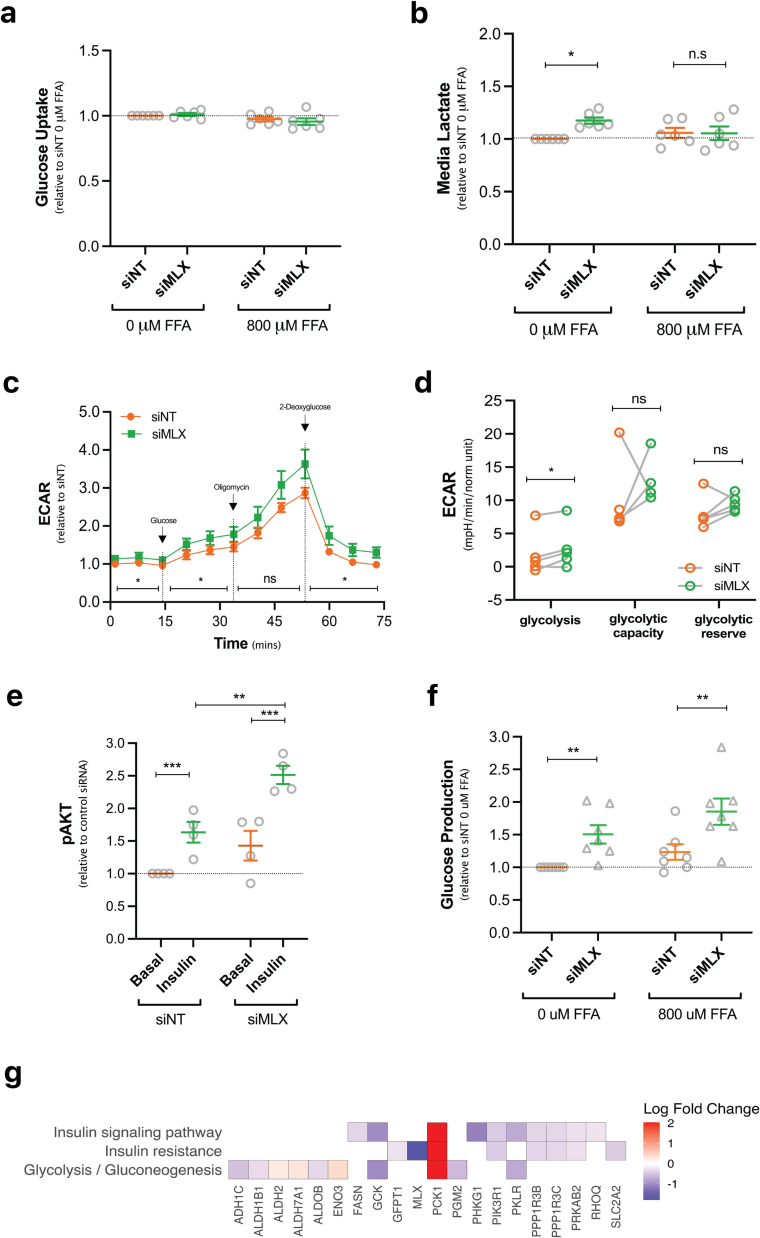
Elevated glycolysis and glucose production following MLX knockdown in PHH. Seven days following transfection with siNT or si*MLX* cell were treated for 72 h with 0 or 800 μM of FFAs before the measurement of **(a)** glucose uptake from and **(b)** lactate secretion into the media. **(c–d)** Cell lysates were used to analyse ECAR using Seahorse XF Glycolysis Stress Test **(e)** and pAKT levels using hTRF. **(f)** Cells were starved overnight and exposed to glucose-free media supplemented with gluconeogenic substrates and forskolin for 5 h prior to media collection and measurement of glucose production. Results are expressed as fold change over siNT or siNT 0 μM FFA and mean + SEM (n = 4–6; each dot represents a donor or independent experiment), *p ≤ 0.05, ** *p* ≤ 0.01, ***p ≤ 0.001. **(g)** Functional data was compared to significantly dysregulated genes (adjusted *p*-value ≤ 0.05) in relevant affected KEGG pathways.

We then sought to investigate the effect of *MLX* knockdown on hepatic glucose metabolism as the liver is a key regulator of systemic glucose levels. Treatment with si*MLX* had no effect on glucose uptake in PHH (Fig. 3a). Once in the cell, glucose can be stored as glycogen or be oxidized via glycolysis. In cells with *MLX* knockdown compared to siNT, glycogen was assessed via two methods and results were inconsistent (data not shown). Thus, the effect of *MLX* expression on glycogen content remains unclear. Media lactate was significantly higher with *MLX* knockdown in the absence of FFA but was not different in the presence of 800 μM FFA (Fig. 3b). This was consistent with the rate of extracellular acid production (ECAR) which showed greater glycolysis with *MLX* knockdown (Fig. 3c–d). In line with higher glycolysis, we found insulin was able to stimulate an increase in pAKT levels in both siNT (*p* = 0.0007) and si*MLX* conditions (*p* = 0.0007) compared to basal (Fig. 3e). We measured glucose production by quantifying glucose secreted into the media after treating cells with gluconeogenic substrates and forskolin for 5 h. *MLX* knockdown was associated with 50 % (*p* = 0.002) and 62 % (*p* = 0.002) greater glucose production compared to siNT in the presence of 0 μM and 800 μM of FFAs respectively (Fig. 3f). We then utilised ^2^H_2_O to explore the effect of MLX knockdown on gluconeogenesis in PHH. There was a trend for higher rates of gluconeogenesis (4.8 ± 0.1 vs 4.0 ± 0.3 μmol/μg protein/h; n = 3, *p* = 0.07) upon *MLX* knockdown. These results are also reflected at the transcriptome level where *PCK1* was the most upregulated gene (S1 Table 2) and significant gene expression changes in relevant pathways (insulin resistance (adjusted *p* = 0.04), insulin signalling pathway (adjusted *p* = 0.02) and glycolysis/gluconeogenesis (adjusted *p* = 3 × 10^−4^)) were observed (Fig. 3g and S1 Table 3).

### 3.4 Humans with *MLX* polymorphism have improved plasma insulin and TG levels

**Fig. 4:**
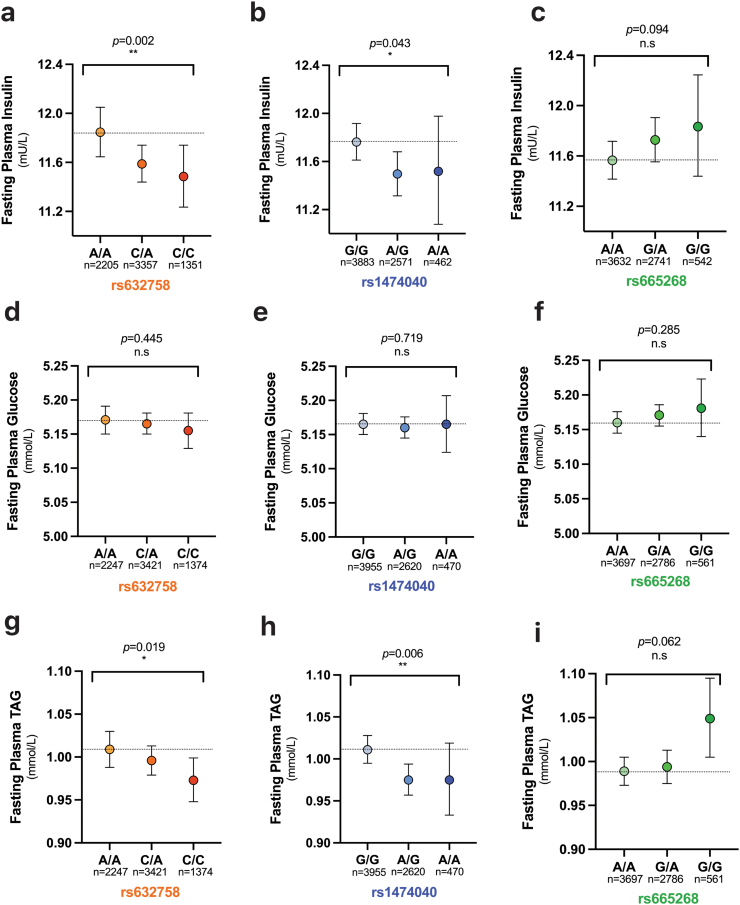
Individuals with polymorphisms in *MLX* have improved plasma insulin and TG levels. Association analyses on plasma biochemistry were performed on imputed genotype data from participants registered with the Oxford Biobank. The relationship between *MLX* genotype and fasting plasma **(a–c)** insulin **(d–f)** glucose and **(g–i)** TG are presented. *p* values are calculated based on a sex combined additive linear regression model adjusted for sex, calculated in the PLINK software and brackets represent the significance of an additive effect across the 3 genotype subgroups within each SNP.

To investigate the metabolic consequences of any genetic variability in the *MLX* gene in the human population, we ran a targeted association analysis for plasma biochemistry and DEXA parameters on imputed genotype data from 7174 healthy individuals registered with the OBB [14]. Twenty-one polymorphic loci were identified in the *MLX* gene region. Across the phenotypes analysed, three independent SNPs were identified: rs665268, rs632758 and rs1474040. Rs632758 is an intronic SNP, rs1474040 a synonymous coding variant (A > A) and rs665268 a nonsynonymous coding missense variant (Q139R) predicted to be deleterious or probably damaging by 4 out of 9 in silico methods for sequence-based prediction of protein function (Supplemental Table 1). In the eQTLgen blood dataset (n = 31,684), *rs1474040-A* (*β(s.e.)* = −0.053 (0.00979), *p* = 5.58E-08), *rs632758-C* (*β(s.e.)* = −0.087 (0.0080), *p* = 1.47E-27) and *rs665268-G* (*β(s.e.)* = 0.147 (0.0089), *p* = 2.16E-61) alleles were significantly associated with *MLX* expression [43]. HOMA-IR was significantly lower in mutant allele carriers in the rs632758 group, and higher in the rs665268 group, but did not differ significantly in the rs1474040 (Table 1). The observed differences in HOMA-IR were largely driven by significant (*p* ≤ 0.05) differences in fasting plasma insulin, with concentrations being lower in mutant allele carriers in the rs632758 and rs1474040 groups, and, although not significant, higher in mutant allele carriers in the rs665268 group (Fig. 4a–c). There were no differences across *MLX* genotype subgroups in fasting plasma glucose concentrations (Fig. 4d–f). Plasma TG concentrations followed a similar pattern to plasma insulin across the *MLX* genotype subgroups, with them being significantly lower in individuals with the rs632758-C or rs1474040-A variant and it tending to be higher in individuals with the rs665268-G variant (Fig. 4g–i). The rs665268-G variant was primarily associated with an increase in DEXA-derived fat mass, mainly driven by an increase in upper body fat mass with a significant association with android fat mass and android/gynoid ratio (Table 1). These associations with fat mass were not reflected in the anthropometric waist and hip measures. Although we found no difference across the *MLX* genotype subgroups in the adipose tissue insulin resistance index (adipoIR), nor fasting plasma NEFA concentration, we did observe fasting plasma glycerol concentrations to be modestly (*p* < 0.05) increased in rs665268-G carriers when compared to the other *MLX* genotype subgroups.

We had the opportunity to explore the effect of the three *MLX* genotypes on IHTG content and fasting hepatic DNL and gluconeogenesis using previously published data [15], [16], [17], [18], [19] along with data from ongoing studies where IHTG content and hepatic DNL had been assessed and where samples were available for the measurement of hepatic gluconeogenesis [22]. We found that IHTG content was not notably different between wildtype and heterozygous and homozygous variant carriers across the three SNPs (data not shown). Although gluconeogenesis did not differ between the respective genotype groups (data not shown), we found hepatic DNL was significantly lower in rs632758-C allele carriers compared to wild type group (10 ± 1.2 % AA (n = 80) vs 5.8 ± 0.6 % CA (n = 42) vs 6.5 ± 0.7 % CC (n = 11), *p* = 0.001).

## 4 Discussion

MLX is a functional heterodimeric partner of ChREBP and MondoA and thus has the potential to influence substrate utilization, particularly glucose. Studies have focused on the role of MLX in regulating glucose partitioning into several pathways, such as oxidation (glycolysis), storage (glycogen), and conversion to fatty acids (DNL) [44]; on the basis of these observations it has been suggested to have therapeutic potential for NAFLD and T2D [44]. Rodent models with genetic modulation of *MLX* or its partners have shown promise in alleviating IHTG accumulation but the effect on insulin sensitivity and glucose metabolism remains unclear [6], [7], [10], [45]. As human data is sparse, we investigated the role of *MLX*-regulated lipid and glucose metabolism in PHH and a cohort of healthy humans with and without *MLX* SNPs.

By performing siRNA-mediated knockdown of *MLX* in PHH, we found IHTG accumulation decreased and this was primarily explained by enhanced fatty acid oxidation and, to a lesser extent, attenuated DNL; TG secretion was not significantly altered. Glucose production was significantly elevated after *MLX* knockdown, regardless of the presence of FFAs and despite higher pAKT expression and greater levels of glycolysis and ketogenesis. Therefore, *MLX* knockdown in PHH appears to shift cellular metabolism away from anabolic processes (such as DNL and TG synthesis), and towards catabolic processes (such as glycolysis, fatty acid oxidation and ketogenesis).

We compared our in vitro data with human in vivo data where we identified 3 SNPs of interest: rs665268, 14704040, rs632758. Our observations from the in vitro hepatocyte data were consistent with fasting plasma biochemical measurements from individuals with *MLX* rs1474040 A/A and rs632758 C/C genotypes, where the homozygous variant for both SNPs was associated with lower plasma insulin and subsequently HOMA-IR, while plasma glucose was not different. Thus, it could be postulated that in the absence of *MLX*, hepatocytes will shift from a state of energy storage to a state of energy utilization.

Intracellular glucose can be stored as glycogen or be converted to fatty acids via DNL. Mice lacking functional hepatic MLX are reported to have decreased expression of genes encoding enzymes involved in *DNL* (*ACC1* and *FASN*) [9]. We assessed DNL using stable-isotope methodology and found the contribution of ^13^C-labelled sugars to DNL-derived fatty acids was reduced with *MLX* knockdown in PHH while in humans, using heavy water, we observed a significantly lower contribution of DNL-derived fatty acids to VLDL-TG in heterozygous or homozygous variant carriers of the rs632758 SNP, but not carriers of the rs1474040 and rs665268 SNPs. Although we did not assess the contribution of ^13^C-labelled sugars to DNL-derived fatty acids in humans, it could be speculated that it would be lower in variant carriers of the rs632758 SNP. The lack of difference between groups in plasma glucose concentrations suggests that any excess substrate was either utilised by other pathways in the liver or disposed in other tissues such as skeletal muscle and adipose.

Previously, we have found when we have decreased hepatic DNL through supplementation with n-3 fatty acids, fasting (and postprandial) plasma glucose concentrations significantly increased [16]. Thus, it is plausible that when DNL is constitutively downregulated, there is adaption by other pathways. In contrast to the in vitro data, lower fasting hepatic DNL in humans did not result in lower IHTG content across the groups. As other fatty acid sources such as adipose tissue-derived fatty acids contribute to a greater extent to IHTG synthesis [46], it is plausible the difference in fasting hepatic DNL was not sufficient to detect subtle changes in IHTG content. Additionally, we did not measure VLDL-TG production and/or secretion which may differ between the specific genotypes.

In contrast to most [6], [7], [9], but not all [10], [45], previously published studies on ChREBP or *MLX* modulation where downregulated expression of glycolytic genes has been reported, we observed an increase in glycolysis with si*MLX* in the absence of FFAs, but a lack of change in media lactate between si*MLX* and siNT-treated PHH in the presence of FFAs. An explanation for these differential effects of *MLX* or *ChREBP* knockdown on glycolysis in our findings and across rodent studies may be due to differences in the availability of FFAs. A recent study performed in human macrophages found that MLX-ChREBP and MLX-MondoA localized to lipid droplets in cells that accumulate fatty acids and this partitioning reduced their availability for transcriptional activity [47], [48]. Thus, it would seem both siRNA-mediated knockdown and the presence of FFAs can downregulate MLX function, which may in part be explained by the presence of lipid droplets which have been shown to sequester MLX, reducing its transcriptional activity. Availability of FFAs may also interfere with the role that MLX and ChREBP play in substrate switching and utilization. In mice with global ChREBP knockout fed a high starch diet (6 % fat), adipose tissue fatty acid synthesis was impaired and was associated with a 2-fold decrease in plasma FFAs, which led to reduced rates of hepatic fatty acid oxidation and ketogenesis, and increased glycolysis [45]. Without dietary or adipose tissue-derived FFAs, global ChREBP knockout mice have reduced substrate for fatty acid oxidation, thereby promoting pyruvate and lactate oxidation [45]. This is further supported by elevated glycolysis observed in fasted liver-specific ChREBP knockout mice; there are no studies that have studied liver-specific MLX or ChREBP knockout mice fed a high-fat diet. In-line with these findings, *MLX* knockdown was associated with greater fatty acid oxidation and ketogenesis in the presence of FFAs and greater glycolysis in the absence of FFAs.

Glucose homeostasis is maintained during fasting by the breakdown of glycogen and/or the synthesis of new glucose from gluconeogenic precursors. High gluconeogenesis is often considered a marker of hepatic insulin resistance however, in the fasting state, this could be an adaptation to an attenuation in glycogenolysis (due to glycogen depletion). We were not able to reliably measure glycogen content in PHH however, *PCK1* was the most upregulated gene in the transcriptome, suggesting a potential effect on gluconeogenesis, though we observed no difference in fasting gluconeogenesis across genotypes in humans. A lack of functional Mlx has been previously shown to increase PEPCK expression in drosophila via inhibition of *cbt*, however there is no human ortholog [49]. In our exploratory data, we observed that cells with *MLX* knockdown had higher rates of gluconeogenesis compared to siNT treated cells. Interestingly, we observed greater pAKT levels in the higher glucose-producing si*MLX*-treated hepatocytes, highlighting the relative importance of insulin signalling vs substrate availability in controlling hepatic glucose production [50]. Continued glucose production despite intact insulin signalling may be explained by attenuated levels of DNL which can lead to an increase in TCA intermediates, namely citrate, and has been shown to enhance gluconeogenesis through fructose 1,6-bisphosphatase [51]. An inverse relationship between DNL and gluconeogenesis has been previously reported by Benhamed et al. [6]. Mice with *ChREBP* overexpression had lower blood glucose following insulin and pyruvate tolerance tests, despite higher expression of DNL genes and IHTG accumulation; however, the effect on pAKT expression was in contrast to our study [6]. In humans, although we found no difference in plasma glucose concentrations across all genotypes, we found fasting plasma insulin concentrations were significantly lower in individuals with *MLX* SNPs rs1474040-A and rs632758-C compared to heterozygote and homozygous wild type carriers, which is consistent with greater pAKT expression in si*MLX*-treated hepatocytes.

Our study has several strengths, including the use of PHH to assess key metabolic pathways using stable isotope tracers. Further, we were able to investigate associations between *MLX* SNPs and fasting plasma biochemistry from the OBB, a nearly 8000-strong cohort of healthy volunteers with available genotype and plasma biochemistry data. We were also able to explore the associations of *MLX* SNPs with liver fat content, DNL and gluconeogenesis in a small cohort of participants who previously undertook a study involving the consumption of heavy water.

Our study is not without limitations. Although we have presented targeted analysis from a large human cohort, we were not able to ascertain whether the SNPs we investigated were causal for the phenotypes measured or their liver-specific effects. Moreover, despite associations with increased mRNA levels and a predicted to impact on protein structure, the net effect of rs665268-G on MLX function is unclear. Future work focused on replicating these results in other larger cohorts could address this. Additionally, in the human studies, diets were not standardized prior to assessing biochemical parameters. Given the role of *MLX* in glucose partitioning, it would be of interest to determine the effect of a perturbation such as a high carbohydrate or high fat diet on relevant hepatic pathways. Further, we have only presented data from the fasting state however the in vitro data suggests that the effect of *MLX* modulation on liver metabolism depends on the availability of both glucose and fatty acids which would be altered in the postprandial state. Whether MLX knockdown promotes fatty acid oxidation via its role as a transcription factor, a lipid droplet protein or another mechanism warrants further study*.* Although we were not able to determine the influence of *MLX* expression on hepatic glycogen content in PHH and humans across the spectrum of genotypes presented here, it would be of interest to do so. Moreover, ChREBP has been reported to have a role in the regulation of cell cycle and proliferation and therefore, it is plausible that knockdown of MLX may promote cell cycle progression. However, as PHH do not proliferate, we were not able to explore this hypothesis in our model.

## 5 Conclusion

We have demonstrated using human in vitro and in vivo models that MLX functions at the intersection of anabolic and catabolic processes and when knocked down, cellular metabolism shifts from DNL and TG synthesis and secretion towards fatty acid oxidation and ketogenesis and shifts glucose partitioning. Typically, IHTG content is associated with increased HOMA-IR, DNL and plasma TG however, we observed a dissociation between these parameters. It is plausible that extrahepatic tissues play a more substantial role in metabolic homeostasis in individuals with certain *MLX* SNPs. Taken together, within hepatocytes, MLX plays a key role in regulating substrate utilization and may alter the composition of liver nutrients which may have effects on metabolic health.

## Financial support statement

This work was supported by a Novo Nordisk Postdoctoral Fellowship run in partnership with the University of Oxford (SRN); the 10.13039/501100000274British Heart Foundation (Fellowship FS/15/56/31645 and FS/SBSRF/21/31013 to LH), the Oxford British Heart Foundation Centre of Research Excellence (RE/18/3/34214), the 10.13039/501100000268Biotechnology and Biological Sciences Research Council (BB/N005600/1 and BB/N015665/1) to LH and the Biotechnology and Biological Sciences Research Council Institute Strategic Programme Food Innovation and Health (BB/R012512/1 and its constituent project BBS/E/F/000PR10347) to LH.

## CRediT authorship contribution statement

Study concept and design: SRN, MAR, LH.

Acquisition of data: SRN, EJL, TM, LCL, SHLA, AC, DJD, MJN.

Analysis and interpretation of data: SRN, EJL, TM, MJN, LC, CM, MAR, LH.

Drafting and revision of the manuscript: SRN, EJL, MAR, LH.

All authors read and approved the final manuscript.

## Declaration of competing interest

EJL, TM, LCL, SHLA, AC, LC, CM and MAR are employees of Novo Nordisk Ltd. All other authors have no conflicts to declare.

## Appendix A Supplementary data

S1 Tables. Lists of differentially expressed transcriptome following *MLX* knockdown in PHH. (Table 1) Top 50 genes with the most statistically significant binding of *MLX* within 1500 base pairs of their transcription site was determined using publicly available CHIP-seq data targeting *MLX* in HepG2 cells (Table 2). After filtering out low detected genes, differentially expressed genes were identified (Table 3). A pathway enrichment analysis was performed using KEGG gene sets to determine the top differentially regulated pathways. Image 1

S2 Table. In silico predicted impact of rs665268 (p.Q139R) on protein function. Image 2
