# Supplementary material for: MLX plays a key role in lipid and glucose metabolism in humans: Evidence from in vitro and in vivo studies
Source: Metabolism. 2023 Jul;144:155563. doi: 10.1016/j.metabol.2023.155563 (PMC10687193; doi:10.1016/j.metabol.2023.155563)
Supplement: Supplementary file 2 — S2 Table. In silico predicted impact of rs665268 (p.Q139R) on protein function. [file mmc2.docx]

# Supplemental Table 2.

| **Tool** | **Prediction** | **Reference** |
| --- | --- | --- |
| SIFT | Deleterious (0.03) | 1 |
| PolyPhen | Probably damaging (0.982) | 2 |
| CADD_PHRED | 22.1 | 3 |
| LRT_pred | Deleterious (0.8433) | 4 |
| MetaLR_pred | Tolerated (0.00011) | 5 |
| MutationTaster_pred | Probably Harmless | 6 |
| MutationAssessor_pred | Medium | 7 |
| REVEL_score | 0.364 | 8 |
| fathmm-MKL_coding_pred | Deleterious | 9 |

**References**

1. Kumar P, Henikoff S, Ng PC. Predicting the effects of coding non-synonymous variants on protein function using the SIFT algorithm. Nature Protocols 4(8):1073-1081 (2009). doi:10.1038/nprot.2009.86
2. Adzhubei IA, Schmidt S, Peshkin L, Ramensky VE, Gerasimova A, Bork P, Kondrashov AS, Sunyaev SR. A method and server for predicting damaging missense mutations. Nature Methods 7(4):248-249 (2010). doi:10.1038/nmeth0410-248
3. P. , Witten, D.M., Cooper, G.M. and Shendure, J., Kircher, M. CADD: predicting the deleteriousness of variants throughout the human genome. Nucleic Acids Res. 47:D886–D894 (2018). doi:10.1093/nar/gky1016.
4. Chun S, Fay JC. Identification of deleterious mutations within three human genomes. Genome Res. 2009 Sep;19(9):1553-61. doi: 10.1101/gr.092619.109. Epub 2009 Jul 14. PMID: 19602639; PMCID: PMC2752137
5. Reva B, Antipin Y, Sander C. Predicting the Functional Impact of Protein Mutations: Application to Cancer Genomics. Nucleic Acids Research (2011).
6. Schwarz JM, Rödelsperger C, Schuelke M, Seelow D. MutationTaster evaluates disease-causing potential of sequence alterations. Nat Methods. 2010 Aug;7(8):575-6. doi: 10.1038/nmeth0810-575. PMID: 20676075.
7. Gnad F, Baucom A, Mukhyala K, Manning G, Zhang Z. Assessment of computational methods for predicting the effects of missense mutations in human cancers. BMC Genomics. 2013;14 Suppl 3(Suppl 3):S7. doi: 10.1186/1471-2164-14-S3-S7. Epub 2013 May 28. PMID: 23819521; PMCID: PMC3665581.
8. Ioannidis, N.M., Rothstein, J.H., Pejaver, V., Middha, S., McDonnell, S.K., Baheti, S., Musolf, A., Li, Q., Holzinger, E., Karyadi, D., et al. REVEL: An Ensemble Method for Predicting the Pathogenicity of Rare Missense Variants. Am. J. Hum. Genet. 99, 877-885. (2016). doi:10.1016/j.ajhg.2016.08.01
9. Hashem A. Shihab, Mark F. Rogers, Julian Gough, Matthew Mort, David N. Cooper, Ian N. M. Day, Tom R. Gaunt, Colin Campbell, An integrative approach to predicting the functional effects of non-coding and coding sequence variation, Bioinformatics, Volume 31, Issue 10, May 2015, Pages 1536-1543,  https://doi.org/10.1093/bioinformatics/btv009
